# Leadership Trends in Shoulder and Elbow Surgery Fellowship Directors: A Cross-sectional Study

**DOI:** 10.5435/JAAOSGlobal-D-21-00266

**Published:** 2022-04-05

**Authors:** Aman Chopra, Melissa A. Wright, Christopher S. Klifto, Oke Anakwenze, Anand M. Murthi

**Affiliations:** From the Georgetown University School of Medicine, Washington, DC (Mr. Chopra); the Department of Orthopaedic Surgery, (Dr. Wright, Dr. Murthi), MedStar Union Memorial Hospital, Baltimore, MD (Dr. Wright, and Dr. Murthi); and the Department of Orthopaedic Surgery (Dr. Klifto, Dr. Anakwenze), Duke University, Durham, NC (Dr. Klifto, and Dr. Anakwenze).

## Abstract

**Introduction:**

We aimed to describe the demographic and professional backgrounds of current shoulder and elbow fellowship directors.

**Methods:**

The American Shoulder and Elbow Surgeons (ASES) 2021 to 2022 Fellowship Directory was reviewed to identify the 31 ASES-recognized US fellowship programs. Demographic and other data were obtained through an electronic survey and publicly available online resources from February 28, 2021, to March 5, 2021.

**Results:**

Of the 31 fellowship directors, 97% (30) identified as male and 74% (23) as White, the mean age was 53 ± 7 years, and the mean Scopus h-index was 24.2 ± 13. Almost all (95%) held ASES committee leadership appointments in at least one committee. The mean time from completion of most recent fellowship to fellowship director appointment was 7.3 ± 6 years. About two-thirds of fellowship directors trained at one of five fellowship programs: Columbia University (n = 7), California Pacific Orthopaedics (n = 4), Washington University in St. Louis (n = 3), Mayo Clinic (n = 3), and Hospital for Special Surgery (n = 2).

**Discussion:**

ASES fellowship directors share similar demographic and professional characteristics with high levels of research productivity and involvement in orthopaedic societies. There is a lack of diversity in shoulder and elbow fellowship directors, highlighting a need for priority consideration of this disparity by leaders in the field.

Shoulder and elbow surgery is a relatively small but distinctive and growing subspecialty of orthopaedic surgery with strong fellowship programs. The fellowship directors who lead these programs are a special set of individuals who devote their time, knowledge, and skill sets to develop well-trained physicians. They play an important role in maintaining teaching quality and defining the specialty for the future. There may be certain innate qualities in these physician leaders who find satisfaction in enhancing the education of others, and these qualities may be specific to each field.

The qualities and characteristics of fellowship directors in other orthopaedic subspecialties have recently been reported. Studies of spine, adult reconstruction, and sports medicine fellowship directors have reported a predominance of White males, a high level of research productivity, and even common residency and fellowship training programs.^1-3^ Fellowship directors have a unique role in orthopaedic specialties in that they are largely responsible for the quality of fellow education, training program design, and trainee job placement. They are not just educators; they are leaders shaping the future of the specialty. Understanding the characteristics of fellowship directors may provide insight into what is valued by the field itself and highlight potential areas for change in the selection and retention of individuals in this important position.

The characteristics of fellowship directors in shoulder and elbow surgery have not yet been reported. We therefore aimed to describe the demographic and professional characteristics of current shoulder and elbow fellowship directors to determine commonalities in their backgrounds that may be relevant for the future of the specialty.

## Methods

The American Shoulder and Elbow Surgeons (ASES) Fellowship Directory for 2021 to 2022 was reviewed for the 31 ASES-recognized shoulder and elbow fellowship programs in the United States. The fellowship director was identified for every program. Demographic, educational, and professional data for all directors were obtained through publicly available sources, which included uploaded current curriculum vitae, institutional biographies, professional media accounts (Doximity and LinkedIn), and Scopus/Web of Science database. In addition, a 12-question survey was sent directly to the institutional e-mail address of all fellowship directors to obtain appointment information that was not consistently available through a public source. All surveys were electronically distributed and completed between February 28, 2021, and March 5, 2021.

We used public sources to collect age, sex, race, graduate degrees, past residency and fellowship training programs, year of residency and fellowship graduation, fellowship types, secondary completed fellowships, if any, and the current institutional setting. Institutional biographies were used to confirm the sex and race of fellowship directors based on pronoun choice and other self-identifying terms used. Individuals were directly contacted through e-mail to clarify any discrepancies. Fellowship director race/ethnicity was described as White, African American, Asian, or Hispanic/Latino based on the collected self-report data and categories used in previous studies of the demographics of orthopaedic leaders.^1-3^ An h-index was determined for each fellowship director by accessing (March 5, 2021) the Scopus/Web of Science database, a search engine that scans peer-reviewed scientific literature with a citation tracking component. The h-index is a metric used to evaluate the total effect of an author's research output through analysis of the number and quality of publications and the number of citations generated per publication. After 20 years of scientific activity, an h-index value of 20 or more is considered to represent a successful researcher, and a value of 40 or more is considered to represent an outstanding researcher.^4^ The survey data included the year the fellowship director was hired by the current institution, time between fellowship completion and fellowship director appointment, academic title at the time of fellowship director appointment, tenure track, past or present institutional titles, board certifications, national society participation, and past or present ASES committee position or positions. Fellowship directors were given three options in the survey regarding their tenure track status at the time of appointment to fellowship director: tenure track, not tenure track, or none (no tenure track offered at the institution). Because no shoulder and elbow surgery board certification exists, the survey recorded any additional subspecialty board certifications for hand surgery or sports medicine. Descriptive statistical analysis was conducted using Microsoft Excel 2021 (version 16.46).

## Results

The fellowship directors from all 31 ASES-recognized shoulder and elbow fellowship programs were included in this study. The 31 institutions with fellowship programs included 16 academic institutions (52%), five private healthcare systems (16%), and six private practice settings (19%). Of 31 fellowship directors, most were male (97%) and White (74%) (Table 1). Seven fellowship directors were Asian (23%). There were no African American fellowship directors, and there was only one Hispanic/Latino fellowship director. The mean age was 52.8 ± 7 years. Four fellowship directors (13%) held an additional graduate degree (MS, MPH, MBA, or PhD).

**Table 1:** Demographics of Current Shoulder and Elbow Surgery Fellowship Directors

Characteristic	Data
Age, yrs, mean ± SD (range)	52.8 ± 7 (40-65)
Sex, n (%)	
Male	30 (97)
Female	1 (3)
Race/ethnicity, n (%)	
White	23 (74)
African American	0 (0)
Asian	7 (23)
Hispanic/Latino	1 (3)

Current fellowship directors were hired between 1990 and 2016, and the mean time from completion of most recent fellowship to institutional appointment as fellowship director was 7.3 ± 6 years (Table 2). At the time of appointment as fellowship director, the most common academic title was associate professor (Table 3). Nine FDs (29%) did not hold an academic title. Current FDs have been in their position for an average of 9.0 ± 6 years. Most directors had a current academic title, with 14 full professors (45%), eight associate professors (26%), and one assistant professor (3%). Eight fellowship directors (26%) were on an academic tenure track, and 11 (35%) were on an academic nontenure track. Fellowship directors had held or were currently holding additional leadership titles within their institutions, including division chief or director (16, 52%), department vice chair (8, 26%), residency program director (4, 13%), and department chair (2, 6%) (Table 3). Twenty-four fellowship directors had no specialty board certification (77%), whereas five were board certified in sports medicine (16%) and two were board certified in hand surgery (6%).

**Table 2:** Shoulder and Elbow Surgery Fellowship Director Educational Background

Characteristic	Data
Graduation yr, mean ± SD (range)	
Residency	2000 ± 7 (1987-2012)
Fellowship	2001 ± 7 (1989-2013)
Years from fellowship to appointment, mean ± SD (range)	7.3 ± 6 (0-19)
Years in position	9.0 ± 6 (1-24)
Location of position, n (%)	
Own residency training site	9 (29)
Own fellowship training site	5 (16)
Own residency and fellowship training site	2 (6)
Primary fellowship, n (%)	
Shoulder and elbow	23 (74)
Sports medicine	6 (19)
Hand	2 (6)
Additional fellowship experience, n (%)	9 (29)

**Table 3:** Shoulder and Elbow Surgery Fellowship Director Leadership and Professional Background

Faculty status at appointment, n (%)	
Assistant professor	4 (13)
Associate professor	12 (39)
Professor	4 (13)
Other	2 (6)
None	9 (29)
Current faculty status, n (%)	
Assistant professor	1 (3)
Associate professor	8 (26)
Professor	14 (45)
Other	1 (3)
None	7 (23)
Tenure status, n (%)	
Tenure track	8 (26)
Not tenure track	11 (35)
None	12 (39)
Additional title at any time, n (%)	
Department chair	2 (6)
Department vice chair	8 (26)
Residency program director	4 (13)
Division chief/director	16 (52)
Other	11 (35)
ASES committee appointments, n (%)	
0	2 (6)
1	7 (23)
2	9 (29)
3	7 (23)
4	3 (10)
≥5	3 (10)

**Figure 1:**
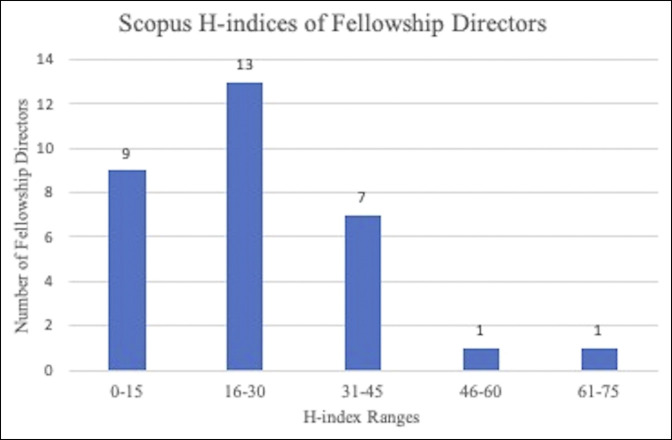
Graph showing a range of Scopus h-index of all shoulder and elbow fellowship directors used to measure research impact and productivity. The Scopus h-index data were retrieved from the database on March 5, 2021.

The mean Scopus h-index for fellowship directors was 24.2 ± 13. The five most impactful directors in clinical research had h-index values of 64, 46, 41, 40, and 39. Nine directors had an h-index value of 0 to 15, 13 had a value from 16 to 30, seven had a value from 31 to 45, and two had a value greater than 45 (Figure 1).

Most of the fellowship directors hold active memberships in the ASES (30, 97%), and 27 (87%) were members of the American Academy of Orthopaedic Surgeons (AAOS). Membership in the American Orthopaedic Society of Sports Medicine (n = 8, 26%) and the Arthroscopy Association of North America (n = 9, 29%) was also common. Fellowship directors held ASES committee leadership appointments in at least one committee (95%), with most involved in multiple committees (Table 3).

**Figure 2:**
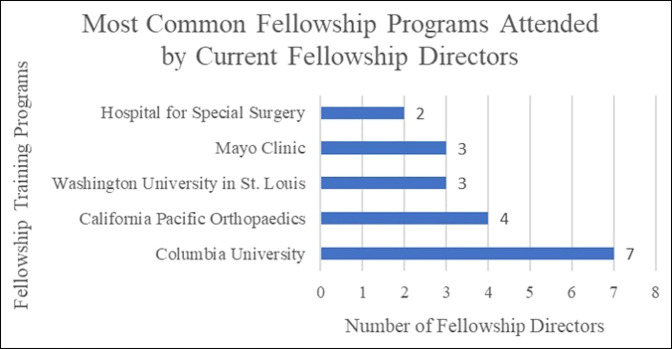
Graph showing the most attended fellowship programs by current fellowship directors in shoulder and elbow surgery. Fellowship programs that produced more than one current fellowship director are shown.

The mean graduation year from residency training for the fellowship directors was 2000 (1987 to 2012) (Table 2). The 31 fellowship directors attended 27 different residency programs. One program, the University of Pennsylvania, graduated three current fellowship directors. Nine fellowship directors (29%) were serving at the same institution in which they completed their residency training. All 31 current FDs completed at least one fellowship with a mean graduation year of 2001 (1989 to 2013). A total of 23 fellowship directors (74%) completed a shoulder and elbow fellowship, 6 (19%) completed a sports medicine fellowship, and 2 (6%) completed a hand fellowship (Table 2). About two-thirds of fellowship directors trained at one of five fellowship programs: Columbia University (n = 7), California Pacific Orthopaedics (n = 4), Washington University in St. Louis (n = 3), Mayo Clinic (n = 3), and Hospital for Special Surgery (n = 2) (Figure 2). Five fellowship directors (16%) were at the same institution in which they completed their fellowship training. Nine (29%) completed a secondary fellowship (Table 2).

## Discussion

This study examined the demographic and professional backgrounds of fellowship directors for the 31 ASES-accredited fellowships to better define the characteristics of a fellowship director in the field of shoulder and elbow surgery. Fellowship directors identified mostly as White males and demonstrated a high level of scholarly productivity, actively participated in academic societies, and tended to graduate from a select set of training programs. These results suggest that research productivity and service to the field are valued in those who reach the role of fellowship director and that these individuals are well suited to develop these qualities in aspiring shoulder and elbow surgeons. The homogeneity of fellowship directors, particularly in terms of sex and race, identifies a critical need to improve the diversity of the field's leaders going forward to benefit the specialty as a whole.

Shoulder and elbow surgeons were appointed to the fellowship director position at a mean age of 52.8 years, nearly identical to the mean age of spine, arthroplasty, and sports medicine fellowship directors at 52.9, 52.6, and 55.4 years, respectively.^1-3^ Shoulder and elbow fellowship directors were also appointed to their role at a similar time after fellowship when compared with other subspecialties. The mean time between fellowship graduation and fellowship director appointment was 8.59 years for spine, 9.55 years for arthroplasty, and 12.8 years for sports medicine.^1-3^ It is clear that multiple years of clinical experience are critical to earning leadership promotions within fellowship education across many subspecialties. In addition to years of clinical experience, prior institutional leadership experience, typically as a division chief or director, was also common among shoulder and elbow fellowship directors.

Academic society involvement and research productivity among shoulder and elbow fellowship directors was high. Fellowship directors had memberships in multiple orthopaedic societies and a high level of committee involvement in the ASES. Society leadership involvement has also been reported to be common among spine fellowship directors, with 13 of 103 directors (13%) having served a full term as president for at least one major spine surgery professional society.^2^ The field of academic orthopaedic surgery places heavy emphasis on research productivity, with one study reporting a median h-index of five across 142 academic orthopaedic surgery departments and 4663 orthopaedic surgeons.^4,5^ Our study demonstrated that shoulder and elbow fellowship directors were quite productive with a mean h-index of 24.2, suggesting that fellowship directors in shoulder and elbow surgery are heavily involved in research and demonstrate a commitment of contributing toward the scientific advancement of their field. Spine and sports medicine fellowship directors had a similar average h-index of 23.8 and 24.1, respectively, and arthroplasty fellowship directors demonstrated an h-index of 16.5.^1-3^

Shoulder and elbow fellowship directors came from a wide variety of residency training programs. A small group of fellowship programs graduated nearly two-thirds of the current fellowship directors in shoulder and elbow surgery. One of these institutions, Washington University in St. Louis, also produced multiple current fellowship directors in both spine and arthroplasty, and another, the Hospital for Special Surgery, produced current fellowship directors in both arthroplasty and sports medicine.^1-3^ These findings suggest that certain programs are associated with producing future leaders in shoulder and elbow surgery and that these same institutions may be associated with producing future leaders across many subspecialties within orthopaedics at the fellowship director position. In addition, 16% of fellowship directors completed their fellowship at the program they now lead. Previous graduates from a program may be more familiar with the program culture and may have existing relationships with faculty members, which is appealing for hiring. However, this practice can perpetuate a lack of diversity among leadership and should be considered carefully.

An important finding was the almost total lack of female representation among shoulder and elbow fellowship directors. This is similar to the representation of women in the role of fellowship director in spine, arthroplasty, and sports medicine.^1-3^ Orthopaedic surgery has the lowest proportion of female residents across all surgical specialties.^6^ Although women represent roughly half of all US medical students, female orthopaedic residents make up less than 1% of all female residents, and only 14% of orthopaedic residents were female in 2017.^6,7^ Although the percentage of female representation in orthopaedic surgery residency has slowly increased to 15.4% in 2019, there is still room for substantial growth.^8^ Increasing the opportunities for women to enter a career in orthopaedic surgery and hold leadership positions is vital to creating future physician role models and leaders who resemble the communities in which they serve and trainees they mentor.

There is also a lack of minority representation among shoulder and elbow fellowship directors. Other studies describing fellowship directors from subspecialties have demonstrated low representation among minority fellowship directors.^1,3^ One study reported that underrepresented minorities made up just 20.2% of orthopaedic surgeons from 2001 to 2008, over half of whom were Asian. Orthopaedic surgery residency programs were found to be markedly less racially and ethnically diverse than other residencies analyzed in that study.^9^ In a study of orthopaedic residents in the United States between 2006 and 2015, 74.5% were White, 12.5% were Asian American, 4.2% were African American, and 4.5% were of Hispanic origin.^10^ Practicing orthopaedic surgeons are even less diverse. A 2018 AAOS member survey reported that 84.7% of members were White, 6.7% were Asian American, 1.9% were African American, and 2.2% were of Hispanic origin.^11^ Asian Americans made up the largest proportion of the minorities represented in both orthopaedic residents and AAOS members, and African Americans and persons of Hispanic origin were the most underrepresented groups, as was also observed in our study.

Directed efforts by individuals, institutions, and leaders of the field will be required to improve diversity among shoulder and elbow surgeons and specifically among fellowship directors. Such efforts have begun with the creation of the ASES Committee on Diversity, Equity, and Inclusion in 2020 to expand leadership opportunities and promote diversity within the shoulder and elbow surgery community. Additional positive measures could include a focus on increasing diversity across all committees in the ASES. Fellowship programs can also focus on developing pathways for female and underrepresented minority orthopaedic surgeons to take on more leadership responsibilities within their programs.

One limitation of this study is that some demographic and professional data were extracted from unverified publicly available institutional biographies, curriculum vitae, and professional social networking platforms. These sources are self-reported and may include errors and incomplete or outdated information. Information from the survey is also subject to possible errors in self-reporting. In addition, race and sex data were presented and analyzed in categories that may fail to capture the complexity of these characteristics. The study design was cross-sectional and thus only represents leadership trends at a single time point. Finally, this study aimed to record objective characteristics of fellowship directors but did not attempt to obtain subjective data such as interpersonal skills, personal connections, and leadership philosophy. These more subjective qualities are difficult to measure but may be more important to what makes an individual not just a fellowship director but a successful mentor and leader in the field.

## Summary

Shoulder and elbow fellowship directors are a distinguished group of individuals who share similar demographic and professional characteristics with high levels of research productivity and involvement in orthopaedic societies. They also share similar educational backgrounds with a select number of fellowship programs producing most of the fellowship directors. The lack of diversity in shoulder and elbow fellowship directors highlights a need for priority consideration of this disparity by leaders in the field, both now and in the future.
